# Neurogenesis and growth factors expression after complete spinal cord transection in *Pleurodeles waltlii*

**DOI:** 10.3389/fncel.2014.00458

**Published:** 2015-01-13

**Authors:** Amira Z. Zaky, Marie Z. Moftah

**Affiliations:** ^1^Biochemistry Department, Faculty of Science, Alexandria UniversityAlexandria, Egypt; ^2^Zoology Department, Faculty of Science, Alexandria UniversityAlexandria, Egypt

**Keywords:** neurogenesis, spinal cord, growth factors, gap replacement, locomotion recovery

## Abstract

Following spinal lesion, connections between the supra-spinal centers and spinal neuronal networks can be disturbed, which causes the deterioration or even the complete absence of sublesional locomotor activity. In mammals, possibilities of locomotion restoration are much reduced since descending tracts either have very poor regenerative ability or do not regenerate at all. However, in lower vertebrates, there is spontaneous locomotion recuperation after complete spinal cord transection at the mid-trunk level. This phenomenon depends on a translesional descending axon re-growth originating from the brainstem. On the other hand, cellular and molecular mechanisms underlying spinal cord regeneration and in parallel, locomotion restoration of the animal, are not well known. Fibroblast growth factor 2 (FGF-2) plays an important role in different processes such as neural induction, neuronal progenitor proliferation and their differentiation. Studies had shown an over expression of this growth factor after tail amputation. Nestin, a protein specific for intermediate filaments, is considered an early marker for neuronal precursors. It has been recently shown that its expression increases after tail transection in urodeles. Using this marker and western blots, our results show that the number of FGF-2 and FGFR2 mRNAs increases and is correlated with an increase in neurogenesis especially in the central canal lining cells immediately after lesion. This study also confirms that spinal cord re-growth through the lesion site initially follows a rostrocaudal direction. In addition to its role known in neuronal differentiation, FGF-2 could be implicated in the differentiation of ependymal cells into neuronal progenitors.

## Introduction

It was in the late 1990s that scientists started to gather interesting information about urodeles. These animals were found to be best suited to study regeneration because they are easy and inexpensive to maintain and breed in the laboratory. Microsurgery is simple, wound healing is rapid and requires very few sutures, and thus a minimal post-operative care is required. Morbidity and mortality are low and results are obtained relatively fast. Their tissues are easy to harvest and process for histological or immuno-cytochemical staining or molecular analysis. On the other hand, known mammalian regenerative mechanisms *in situ* include compensatory hyperplasia (such as in the case of liver and pancreas), and activation of resident stem cells, but not dedifferentiation or trans-differentiation. Moreover, neurogenic ability in most regions of the central nervous system (CNS) is very limited (Goldman, 2004) and the production of new neurons is demonstrated to be almost arrested in adulthood (Hegedus et al., 2007).

The spinal salamander was chosen for this study because it is an ideal model to study not only regeneration but also neuronal and locomotor plasticity. Adult urodele amphibians regenerate their spinal cords following a cut or crush injury, achieving functional recovery. This process is intrinsically appealing (Stocum, 1995, for review) although the time course of regeneration in urodeles depends on the nature of the lesion and on the age of the animal. However, a frequently arising question is whether understanding spinal cord regeneration in urodeles has relevance to spinal cord injury (SCI) in higher (amniote) vertebrates, such as humans (Chernoff, 1996).

Urodeles have two locomotor modes: swimming and over ground stepping, they switch from one mode to the other (functional plasticity) and spontaneously recover locomotion following a severe CNS injury (post lesional plasticity). Regeneration that occurs in embryonic or larval animals involves the formation of substantial numbers of new neurons and is qualitatively different from adult regeneration (Stensaas, 1983; Davis et al., 1989). Different aspects of spinal cord regeneration can be examined at different points in the life cycle, emphasizing neurogenesis or axonal regrowth (Chernoff et al., 2003). It is noteworthy that urodele amphibians are the only tetrapod vertebrates that can regenerate all regions of their spinal cord as adults (Chernoff, 1996).

Despite the fact that a new spinal cord is formed through cellular re-differentiation after tail amputation, body spinal cord regeneration is often a fraction size of the normal spinal cord. It contains few or no neurons, has greatly reduced white matter and has little or no neuropil (Holder and Clarke, 1988). Recovery of locomotion has been demonstrated after transection of brachial, thoracic, lumbar, and tail spinal cord (Stensaas, 1983; Clarke et al., 1988; Davis et al., 1989; O’Hara et al., 1992; Benraiss et al., 1999; Chevallier et al., 2004). Most of these studies use axonal regrowth to measure regeneration as it reflects some degree of rearrangement of the intraspinal circuitry (Becker et al., 2005).

Regenerated axons contribute to the restoration of locomotor initiation below a healed spinal transection. Some of them are brainstem neurons (Chevallier et al., 2004). In some cases, however, recovery of behavior, which had been lost after injury, has been reported in conjunction with the reappearance of distinct types of new neurons (Scharff et al., 2000; Kuscha et al., 2012a,b). Spinal Cord also regenerates through gap replacement. Experimental analyses undertaken in the brain of several vertebrate classes suggested that the constitutive ventricular progenitors serve as a stem cell population that becomes activated to replace the lost tissue upon injury (Font et al., 2001; Zupanc and Clint, 2003; Endo et al., 2007; Parish et al., 2007; Kaslin et al., 2008; Tanaka and Ferretti, 2009; Gonzáles-Granero and Gracia-Verdugo, 2011; Kroehne et al., 2011; Kizil et al., 2012a,b).

One of the main factors described to promote this phenomenon is basic fibroblast growth factor (bFGF), which is known to be an important growth factor that increases neurite extensions in cultured dissociated cells of the urodele spinal cord (Moftah, 2007), in PC12 cells (Rydel and Greene, 1987) and in embryonic stem cell (ESC)-derived neural cells (Lam et al., 2010). It also increases neurogenesis from human neural progenitor cells (NPCs; Nelson and Svendsen, 2006) and stimulates neural cell differentiation (Wilson and Stice, 2006; Moftah et al., 2008; Lam et al., 2010). bFGF is also used to maintain the neural stem cell pool in the mouse brain SVZ (Zheng et al., 2004) and the undifferentiated human ESCs (Xu et al., 2005). Some of the FGFs are probably released from cells when they are damaged (Chernoff, 1996). For example, following injury, ependymal cells upregulate bFGF production both in urodeles (Moftah et al., 2008) and in rodents (Del Bigio, 1995). It is produced by astrocytes in the CNS. It has been suggested that astrocytes orchestrate proliferation in the neurogenic niches by providing FGF-2 among other factors to the neural stem cells. However, for neurogenesis to be completed, progenitors have to stop proliferating, differentiate and survive (Hagg, 2005).

In considering applications to human disease, it has been reported that nestin is not only expressed in mice but also in human neuroepithelial cells suggesting the existence of neural precursors since it is an intermediate filament marker that characterizes embryonic neuroepithelial cells (Lendahl et al., 1990). It was also mentioned that it is expressed in undifferentiated cells during CNS development (Almqvist et al., 2002) and that it loses its expression early during the differentiation of a neural stem cell line (Mellodew et al., 2004). Such findings encourage the possibility that precursor cells from the human CNS may be used in cell replacement or gene therapy strategies directed toward human neurodegenerative disorders.

In the present study, we show that the ependymal cells remained active, filled the gap between both spinal cord stumps and expressed nestin (intermediate filament marker), bFGF, one of its receptors (FGFR2) and neurofilament (NF, adult neurons marker). We therefore describe neurogenesis in adult vertebrate spinal cord after complete transection in the mid-trunk region.

## Material and methods

### Animals

Experiments were carried out as previously described (Moftah et al., 2008). In brief, 25 urodele amphibians (*Pleurodeles waltlii*) were obtained from Blades Biological Ltd (Kent, UK) and kept in aquaria at 19°C. Surgical procedures, handling and housing of the animals were in accordance with protocols approved by the INSERM Ethics Committee and conformed to NIH guidelines.

### Spinalization

Surgery was performed in aseptic conditions under general anesthesia as previously described (Moftah et al., 2008). In short, anesthetized animals were operated by completely cutting the spinal cord between segments 12 and 13. The wound was sutured and wound healing was complete 8–10 day post-operative. Sham-operated animals were exposed to laminectomy but not spinal cord transection.

### *In situ* hybridization

All animal groups were anesthetized and treated for *in situ* hybridization as previously described (Moftah et al., 2008). In summary, spinal cords were exposed by laminectomy then divided into two segments (anterior and posterior) corresponding respectively to the pre- and post-lesional parts of the cord (in spinalized animals). Specimens were immediately but separately frozen by immersion in −50°C isopentane (Merck) without fixation. All samples were stored in embedding medium (Tissue Tek, Sakura) to be sectioned and processed later on.

Spinal cord sections (265 section/region/animal/experiment) were 14 μm thick and processed as described earlier (Landry et al., 2000). Briefly, sections were incubated at 42°C with 0.5 ng of each of the radioactively labeled probes. After hybridization, they were rinsed for four times at 55°C followed by 30 min at room temperature. Radioactivity was revealed by dipping sections into Ilford K5 nuclear emulsion (Ilford, Mobberly, Cheshire, UK), diluted with distilled water in a 1:1 ratio. They were then developed in the Kodak D19 developing solution and fixed in the Kodak 3000 fixative. Sections were then counterstained with 0.25% cresyl violet acetate (pH 4) (Sigma) and mounted in glycerol.

### Oligonucleotide probes

For *in situ* detection of FGF-2 and FGFR2 mRNA, we used the following four fifty-mer oligonucleotide probes (Eurogentech, Seraing, Belgium) based on previously published gene sequences (Zhang et al., 2000; Moftah et al., 2002) respectively.

*FGF-2*:

- 5^′^GTTGATCCGCAGAAAGAAGCCCCCGTTCTTGCAGTACAGCCTCTTGGGTC3^′^
- 5^′^TTCAGCGCCATAAGCCTGCCGTCATCCTTCATAGCGAGATAGCGGTTTGC3^′^

*FGFR2*:

- 5^′^GGAAATGGACCAGGAACTTACTCTAAAAAGATGGTCAGCTGGGATTCGGG3^′^
- 5^′^CCTGGTGTCAGGGTAGCTAGGTGAATACTGCTCCAGAGGTCCGCTGAGGT3^′^

Probes were chosen from regions presenting few homologies with related mRNA sequences and were checked against the GenBank database.

As previously described (Landry et al., 2000), oligonucleotides were labeled in cobalt containing buffer with ^35^S-dATP (Amersham) to a specific activity of 1–4 × 10^9^ cpm/μg and purified by ethanol precipitation.

### Western blots

The presence of FGF2 protein was determined by western blot analysis. All animal groups were anesthetized and treated as previously described (Moftah et al., 2012). In summary, spinal cords were exposed by laminectomy then divided into two segments (anterior and posterior) corresponding respectively to the pre- and post-lesional parts of the cord (in spinalized animals). Specimens were immediately frozen by immersion in –50°C isopentane (Merck) without fixation.

Previously removed and frozen spinal cord portions (*n* = 5) were treated as previously described (Moftah et al., 2012). In short, specimens were separately homogenized in ice cold buffer. Homogenates were centrifuged and 1% Triton X-100 (Sigma) was added. The supernatants were centrifuged for 1 h at 4°C. Protein content was determined using Bradford assay (Bio-Rad, Hercules, CA, USA). Western blots were performed with 40 μg of proteins and repeated five times. Samples were resuspended in Laemmli Buffer (Laemmli, 1970), fractionated by SDS-PAGE using a 10% acrylamide gel and then transferred to PVDF membrane (Bio-Rad). Membranes were washed several times in blocking buffer and incubated overnight at 4°C with rabbit anti-FGF-2 (Chemicon, Temecula, CA, USA) primary antibody (1:200 in blocking buffer: BIORAD, Marnes-la-Coquette, France). Immunoreactivity was detected using anti-rabbit horseradish peroxidase (HRP)-conjugated secondary antibody (Dako) and visualized using enhanced chemiluminescence (ECL) detection system (Cell Signaling, Beverly, MA, USA). Anti-beta-actin was used as control.

### Immunohistochemistry

Anesthetized animals (*n* = 5/experiment) were perfusion-fixed via the ascending aorta. Spinal cord was dissected out and treated as described earlier (Moftah et al., 2012). In summary, blocked sections were incubated (1:1000 in 2,4,6-trinitrotoluene, TNT/bovine serum albumin, BSA) with rabbit anti-nestin (Abcam, Paris, France) and mouse anti-pan neural NF (Sternberger Monoclonals Incorp., Lutherville, MD, USA) primary antibodies. After rinsing in TNT, they were incubated with anti-rabbit Alexa fluor 488-conjugated and anti-mouse Alexa fluor 568-conjugated (Invitrogen, Cergy Pontoise, France) secondary antibodies (1:500 in 1,3,5-Trinitrobenzene, TNB). Slides were then rinsed and mounted.

### Imaging

Slides were examined; bright field light microscopy micrographs were taken using a Zeiss Axiophot 2 microscope (Zeiss, Jena, Germany). Immunostainings were analyzed with a Leica DMR PCS SP2 AOBS confocal microscope (Leica, Heidelberg, Germany) using a 20×, 40× or 63× oil-immersion lens. In all cases, scans were carried out sequentially with the 488 nm and 568 nm lines of the laser. Digital images were optimized for image resolution (300 dpi final resolution), brightness and contrast using Adobe Photoshop 6.0 (Adobe System, San Jose, CA, USA).

### Data analysis

Comparisons between groups (five animals each) were made on sections treated together on the same slides under identical conditions. The number of labeled cells and the labeling intensity were quantified as previously described (Landry et al., 2000). Cellular profiles containing three times more grains than mean background grain densities were considered labeled. Cell profiles were manually outlined as previously described (Moftah et al., 2008). Delineation was based exclusively on staining and not on shape, size or other measurable quantities. The number of silver grains per cresyl violet counterstained cell was counted semi automatically using MetaMorph Offline 6.1 software (Universal Imaging Corporation). Data were expressed as grain density per μm^2^ ± SEM in calibrated photomicrographs. Transmitted light photomicrographs were taken for at least 500 fields (700 μm^2^ each), in each experiment.

Data were imported into a spreadsheet program (Sigma Plot software, Jandel Scientific) that calculated and graphed the density of FGF-2 and FGFR2 mRNA expression. Data were compared using one way ANOVA tests and processed using standard statistical analysis techniques (Sigma Stat software, Jandel Scientific). Differences were considered to be significant when *p* ≤ 0.05.

## Results

### Nestin expression in spinally transected animals throughout spinal cord regeneration

**Figure 1:**
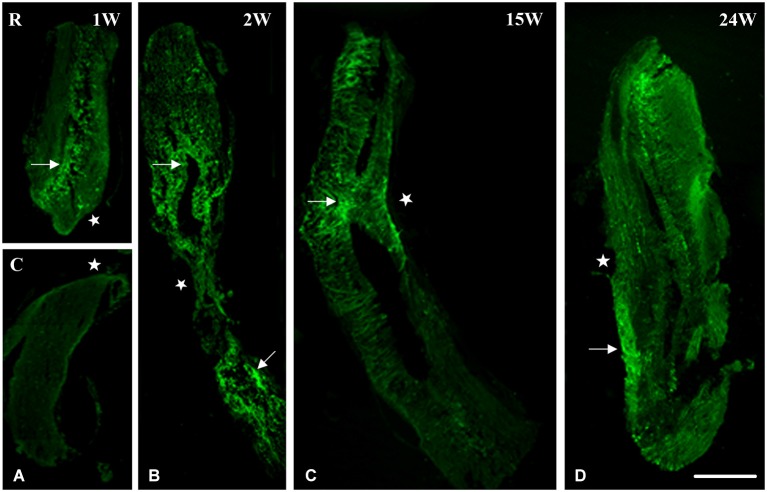
**Imunohistochemical analysis of nestin labeling during spinal cord regeneration. (A)** Separated stumps at 1 week (1 W) post-operatively (R: Rostral; C: Caudal; lesion site denoted by a white star). **(B)** Nestin is seen in the central canal region (arrow), stumps connect and nestin is equally distributed in both parts of the central canal at 2 weeks after transection (2 W: arrows). **(C)** Continuous cord and nestin is mainly expressed at the lesion site after 15 weeks of transection (15 W: arrow). **(D)** Normal spinal cord and few traces of nestin are still obvious 24 weeks post-operatively (24 W: arrow). Scale bar: 200 μm.

Using nestin as a marker for intermediate filaments and thus neurogenesis, we investigated spinal cord regeneration after complete transection in the mid-trunk region. At 1 week after transection, spinal cord was separated into two stumps, a rostral (R) and a caudal (C) one (Figure 1A). Nestin was expressed mainly in the rostral stump around the central canal. This implies that regeneration started in the anterior portion of the spinal cord (Ant SC: the pre-lesional stump) right after transection thus preceding the posterior portion (Post SC), in which nestin is still faint at 1 week after the operation. Starting at 2 weeks post-operatively, a gap replacement is starting to appear, i.e., the gap between the two stumps started to be filled with neuronal progenitors as seen by the equally distributed nestin expression in both parts of the central canal and at the lesion site (Figure 1B). Fifteen weeks after transection; when animals recover their locomotor activity; spinal cord (as seen in Figure 1C) became continuous, completed gap replacement, due to the prominent expression of nestin at the lesion site. After 24 weeks of spinal cord transection, animals recovered both locomotor activities, i.e., stepping and swimming. On the cellular level, we noticed that spinal cord was normal in shape. Nevertheless, few traces of nestin are still obvious (Figure 1D).

### Nestin and neurofilament expression in spinally transected animals 1 week after spinal cord lesion

**Figure 2:**
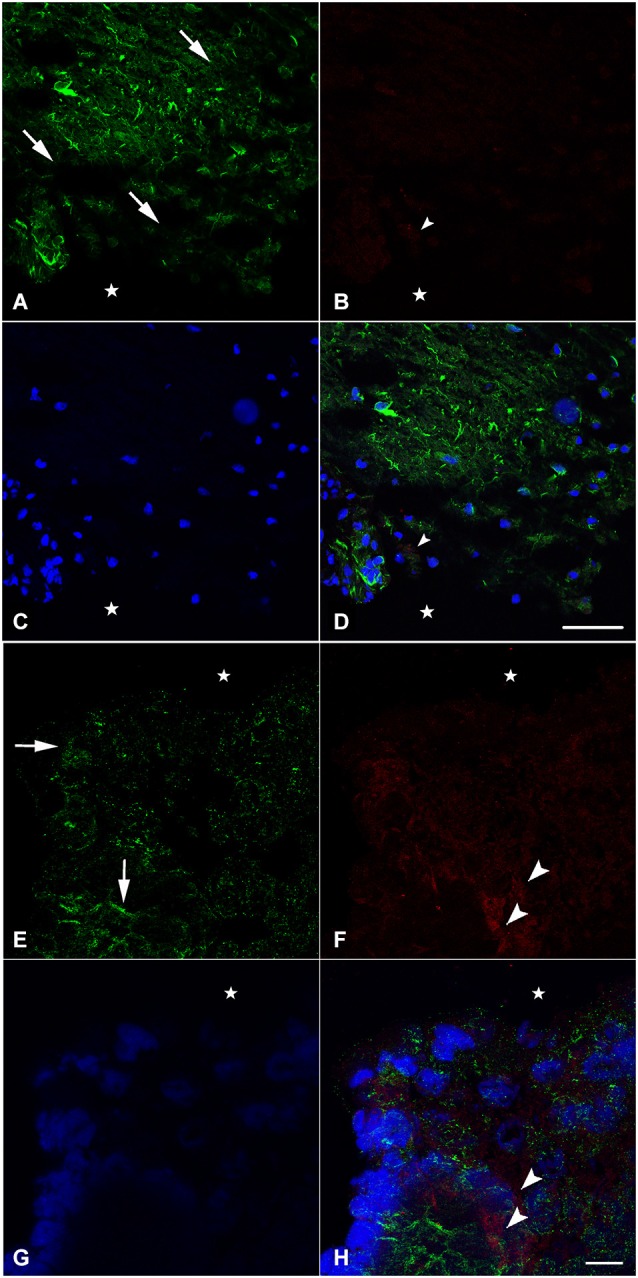
**Imunohistochemical analysis of nestin (green) and NF (red) labeling during spinal cord regeneration in the rostral (A–D) and caudal (E–H) stumps after 1 week of spinal cord transection (stars show the lesion site)**. In **(A)** and **(E)**, nestin expression shows the orientation of neural progenitor cells towards the lesion site in the rostral and caudal stumps of the regenerating spinal cord respectively (arrows show the orientation direction). The neurofilament marker NF is very faint in **(B)** although abundant in **(F)** (arrowheads). **(C)** and **(G)** show the nuclei demarked by Bis-Benzimide while **(D)** is an overlay snapshot showing a single NF-labeled cell (arrowhead) and the abundant nestin labeling surrounding blue nuclei. **(H)** is an overlay snapshot showing NF-labeled cells far from the lesion site (arrowheads) and the abundant nestin labeling closer to the lesion site. Scale bar for **(A–D)** = 120 μm; for **(E–G)** = 20 μm.

In an attempt to get a closer look to the cellular level, we examined the distribution of nestin and NF during spinal cord regeneration. At 1 week after transection, when the animal is completely paralyzed and the spinal cord is separated into a rostral and a caudal stump, we found that nestin expression in the rostral part is prominent next to the lesion site (Figure 2A). The labeling orientation implies a possibility of neuronal progenitor migration from the upper part of the rostral stump to the lesion site in an attempt to replace the missing tissue. Meanwhile, adult neurons have been lost due to transection and the remaining cells disintegrated. We show here that NF, the neuronal marker, is almost absent 1 week post-operatively; a unique neurone is seen near the lesion site (Figure 2B). No colocalization is noticed between NF and nestin (Figure 2D), which implies that nestin is not expressed by neurons but instead is expressed by neuroglial cells as shown in our previous study (Fahmy and Moftah, 2010).

In the caudal stump, however, we found that nestin expression is prominent not only next to the lesion site but also in the deep layers of the lesioned spinal cord (Figure 2E). The labeling orientation implies the same possibility of migration seen in the rostral stump although in a lesser organization. Meanwhile, adult neurons have not been completely lost from the caudal stump after transection. Using NF, we showed that, 1 week after injury, neurons are still abundant in the deep layers of the transected spinal cord (Figure 2F). Although their presence in masses, there still is no colocalization between NF and nestin (Figure 2H), implying the *de novo* formation of neural cells after differentiation of the neural progenitors expressing nestin. When nestin, the marker of neuronal progenitors is more abundant in the rostral stump, NF; the marker of adult neurons is more abundant in the caudal one. This could suggest that the regeneration process follows a rostro-caudal direction.

### Nestin and neurofilament expression in spinally transected animals 6 weeks after spinal cord lesion

**Figure 3:**
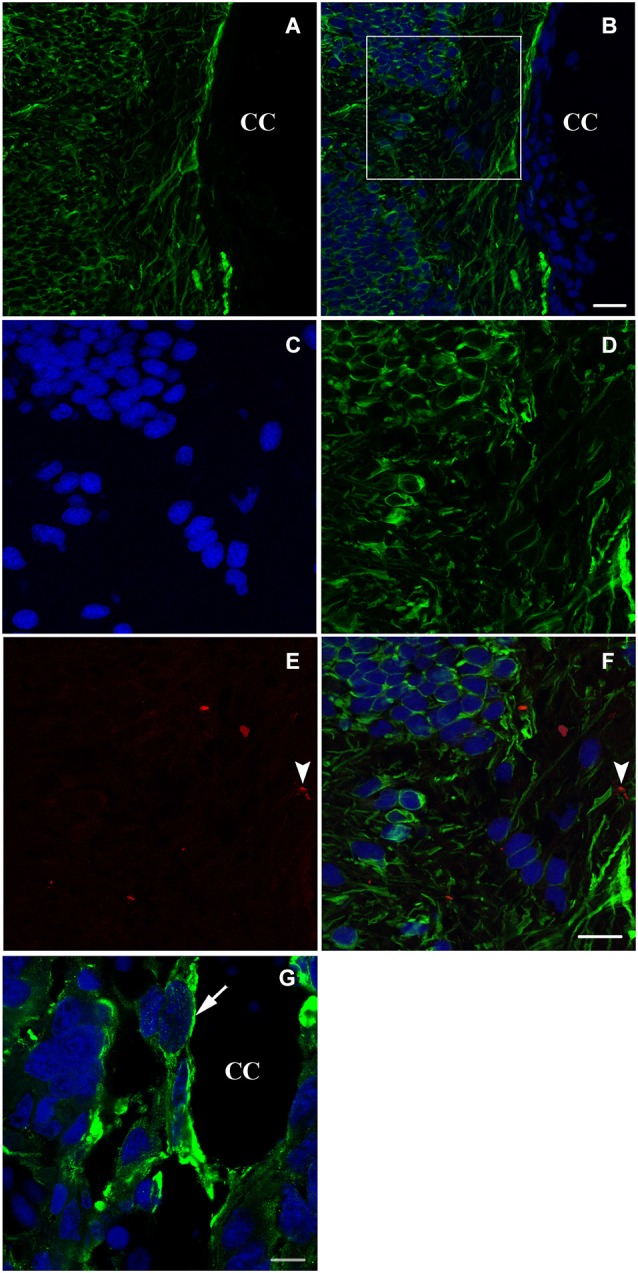
**Longitudinal sections (A–F) and a transverse section (G) showing imunohistochemical analysis of nestin (green) and NF (red in E,F) labeling during spinal cord regeneration after 6 weeks of spinal cord transection**. In **(A)**, nestin expression increases dramatically. **(B)** represents an overlay of nestin and bis-benzimide-labeled nuclei. The white square is magnified in **(C–F). (C)** shows the bis-benzimide-labeled nuclei. **(D)** shows nestin-labeled cells. The neurofilament marker NF is rare in (**E**: arrowhead). **(F)** is an overlay snapshot showing a single NF-labeled cell (arrowhead) and the abundant nestin labeling. In **(G)**, nestin is localizing around the central canal (CC) during spinal cord regeneration, 6 weeks after transection. Scale bar in **(B)** stands for **(A,B)** = 70 μm, in **(F)** stands for **(C–F)** = 40 μm and in **(G)** = 20 μm.

Six weeks after spinal cord transection, when the animal started to recover some of its locomotor activity, the expression of nestin on the cellular level increased dramatically. Cells become more organized and both stumps of the lesioned spinal cord get connected at the transection site; replacing thus the gap created by the operation (Figures 3A–D). Meanwhile, new, very rare adult neurons start appearing next to the central canal as shown in Figures 3E,F.

A more obvious view of nestin expression in cells lining the central canal is seen in a transverse section of the neural axis at the lesion site, 6 weeks after transection (Figure 3G).

### Nestin and neurofilament expression in spinally transected animals 24 weeks after spinal cord lesion

**Figure 4:**
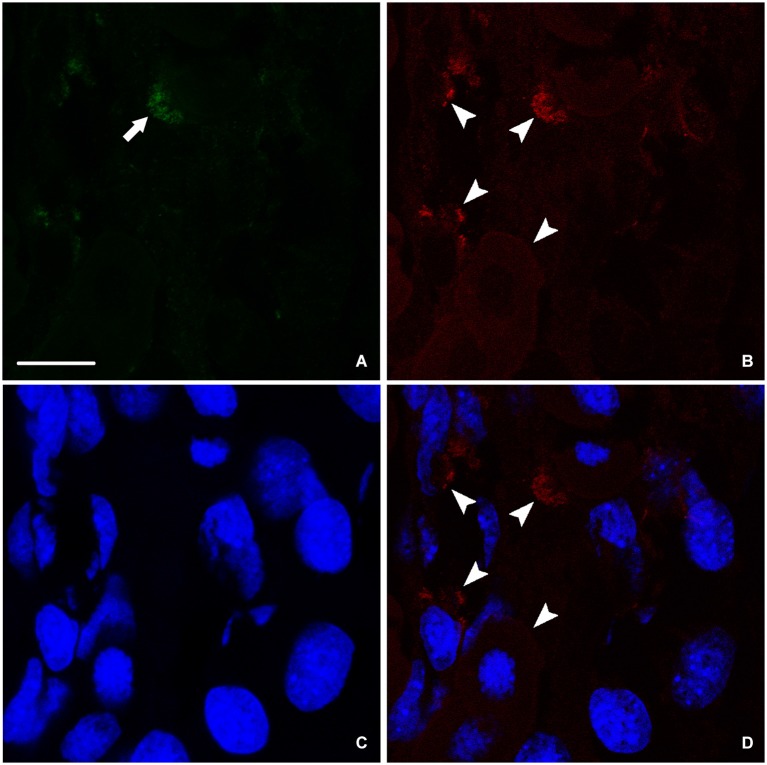
**Imunohistochemical analysis of nestin (green) and NF (red) labeling during spinal cord regeneration after 24 weeks of spinal cord transection. (A)** Faint nestin expression (arrow) situated far from the central canal. NF, arrowheads in **(B)**, is prominent. **(C)** Nuclei demarked by Bis-Benzimide. **(D)** is an overlay snapshot showing neurofilament surrounding the blue nuclei (arrowheads). Scale bar = 25 μm.

At the end of the process of spinal cord regeneration, when the animal completely recovered its locomotor activity after 24 weeks of the operation, sections taken in the lesion site showed the disappearance of the big amount of nestin leaving behind instead a faint labeling (Figure 4A). In the same location, we saw a stronger labeling of NF (Figure 4B), which implies that the newly formed neurons are finally differentiated expressing only NF. This is confirmed by the absence of colocalization between nestin remnants and the neurofilament marker NF as shown in (Figure 4D).

### FGF-2 and FGFR2 mRNA in sham-operated and spinally-transected animals

**Figure 5:**
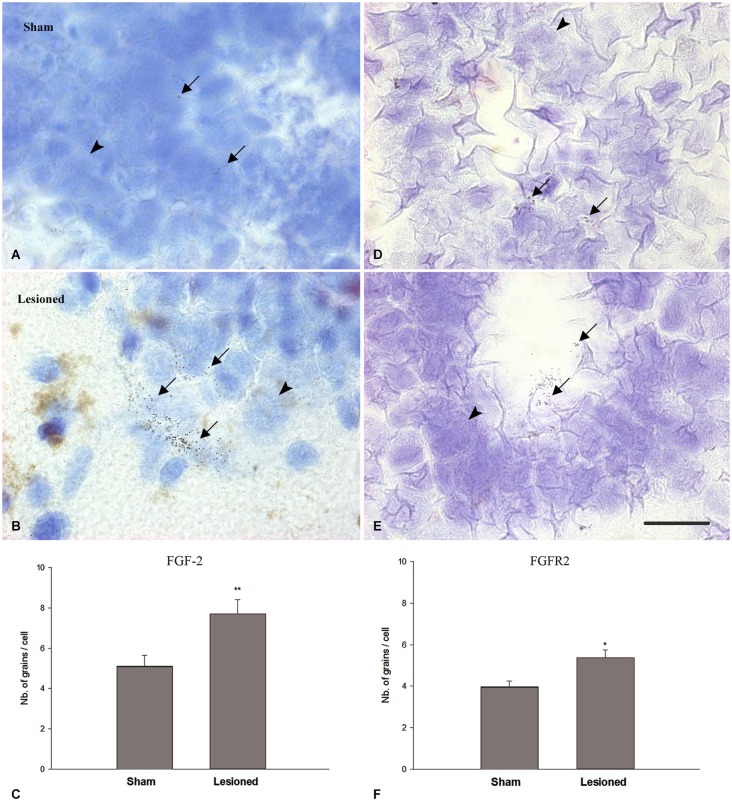
**FGF2 (A–C) and FGFR2 mRNA (D–F) expression (in pre-lesional spinal cord) in sham-operated (A,D) and spinal-transected (B,E) animals at 15 weeks post-operatively. (A)** Localization of FGF-2 mRNA in cross sections of spinal cord showing *in situ* hybridization in the anterior region in sham-operated animals. **(B)** shows anterior spinal cord in lesioned animals. **(D)** Localization of FGFR2 mRNA in cross sections of spinal cord showing *in situ* hybridization in the anterior region in sham-operated animals. **(E)** shows anterior spinal cord in lesioned animals. All sections are dorsoventrally oriented. Arrows in **(A,B,D,E)** show the grains demarking hybridized FGF-2 **(A,B)** and FGFR2 **(D,E)** mRNAs, while arrowheads point at non labeled cells. Grains demarking hybridized FGF-2 mRNAs are more pronounced in number ventrolaterally (arrows in **B**) while FGFR2 grain distribution is mainly seen in the ependymal cells, lining the central canal (arrows in **E**). Scale bar = 40 μm. **(C)** Comparison between FGF-2 mRNA grain density in sham-operated and lesioned animals. In sham-operated animals, the level of grain density is significantly less compared to spinal-transected animals. **(F)** Comparison between FGFR2 mRNA grain density in sham-operated and lesioned animals. A slight increase was observed in lesioned animals compared to shams. Symbols above lesioned animals’ bars indicate their statistical significance compared to sham-operated animals’ bars. (**p ≤ 0.01; *p < 0.05).

In previous studies (Moftah et al., 2008, 2012), we correlated the recovery of locomotor activity with FGF-2 and FGFR2 mRNAs expression. To investigate the relation between the transcriptional activity of this growth factor and its receptor with neurogenesis, we studied the distribution of FGF-2 and FGFR2 mRNA in the same regions we used for nestin immunohistochemistry, by using *in situ* hybridization (Figure 5). Analysis of grain density showed that lesioned animals, 15 weeks after the operation, express high levels of FGF-2 (Figure 5B) and FGFR2 (Figure 5E) mRNA compared to sham-operated animals (Figures 5A,D). FGF-2 mRNA was mainly seen in a ventrolateral position as shown in Figures 5A,B. However, grains demarking FGFR2 mRNA were more prominent in the cells lining the central canal, namely the ependymal cells, as shown in Figures 5D,E. mRNAs grain density quantification showed that FGF-2 significantly increased in lesioned animals 15 weeks post-operatively (*p* = 0.005) compared to the shams (Figure 5C). This increase is consistent as to FGFR2 mRNA but has a smaller magnitude (*p* = 0.03) than its ligand (Figure 5F).

### FGF-2 protein expression in intact, sham-operated and spinally transected animals

**Figure 6:**
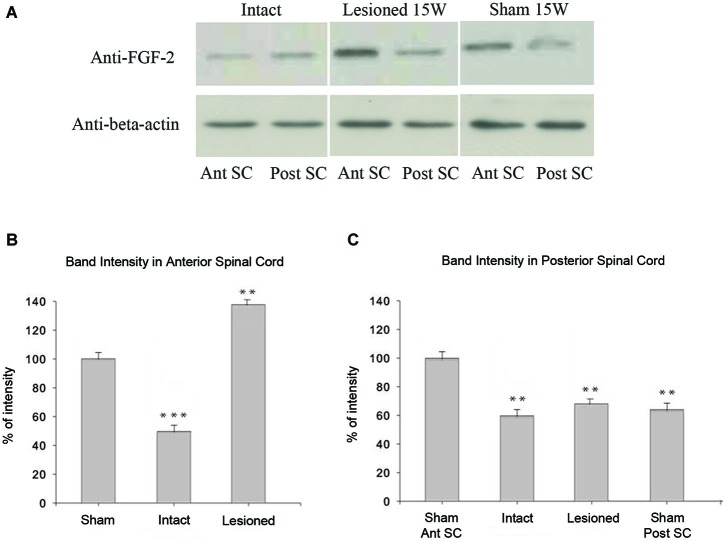
**Immunoblot analysis of FGF-2 expression: (A) shows that a band detected by an anti-FGF-2 antibody is reduced in intact (*n* = 5) pooled anterior parts of spinal cord: Ant SC (upper left band in intact panel) compared to intact (*n* = 5) pooled posterior parts of spinal cord: Post SC (upper right band in intact panel)**. In lesioned animals 15 weeks after transection, the Post SC bands (upper right band in lesioned panel) are less prominent than those of the Ant SC (upper left band in lesioned panel). Sham-operated animals show the same pattern of lesioned ones but at a lesser extent (upper panel of sham). Lower panels show beta-actin in all regions. **(B)** Ant SC band intensity compared to beta-actin bands. Note the maximum intensity in lesioned animals 15 weeks post-operatively. The symbols above each bar indicate the statistical significance with the sham bar. **(C)** Post SC band intensity compared to beta-actin bands. Note the non significant intensity increase in lesioned animals 15 weeks post-operatively. The symbols above each bar indicate the statistical significance with the sham Ant SC bar. (***p ≤ 0.001; **p ≤ 0.01).

In intact animals, western blot assays showed significantly less FGF-2 protein in the anterior part of the spinal cord (Ant SC: corresponding to the pre-lesional portion in transected animals) than in the posterior portion of trunk spinal cord (Post SC; Figure 6A; upper left panel; *p* = 0.01). This was even more evident but inversed in the case of spinally-transected and sham-operated animals where the Ant SC showed more FGF-2 protein expression than Post SC (Figure 6A; upper middle and right panels). Although there was no spinal cord transection in shams, the fact that the skin, muscles and bones were lesioned caused the increase of FGF-2 in the anterior part of the neural axis. Figure 6B shows that the intensity of FGF-2 band of spinally-transected animals is significantly stronger than that of the sham-operated ones 15 weeks post-operatively (*p* = 0.008). This is probably due to spinal cord transection that is absent in shams. Compared to the beta-actin bands, the Ant SC of sham-operated animals was the closest, thus normalized to 100% of intensity. All other bands were compared to it. Figure 6C shows that the intensity of FGF-2 band of spinally-transected animals is slightly but not significantly stronger than that of the intact and the sham-operated ones 15 weeks post-operatively (*p* = 0.01). This implies that FGF-2 protein expression follows a rostrocaudal gradient after complete spinal transection.

## Discussion

Urodele amphibian spinal cord regeneration is a unique experimental system in that urodeles are the only tetrapod vertebrates that are strong regenerators in adulthood (Nicolas et al., 1999). Retention of embryonic character is cited as a property that supports this regeneration process (Chernoff, 1996), but it is not clear to what extent embryonic processes involved in the CNS development is retained or re-expressed. Regenerating urodele spinal cord clearly does not recapitulate the early events of neurulation. However, the example of limb regeneration suggests that some embryonic patterning and differentiative processes would be required to structure and restore function completely (Stocum, 1996; Brockes, 1997; Christensen et al., 2002). In this study, we show some of these embryonic patterning phenomena and differentiative processes, namely, possible cell migration from the deep layers of the lesioned spinal cord towards the central canal (Figures 2A,E) and differentiation of neuronal progenitors expressing nestin into adult neurons expressing NF (Figures 4B,D).

Caudal regeneration studies have shown that multipotent cells are still present in the spinal cord of adult urodeles (Benraiss et al., 1996). The ependymoglial cells of the adult urodele spinal cord are considered pluripotent stem cells that can be recruited for both CNS and peripheral nervous system (PNS) regeneration (Benraiss et al., 1996, 1997). They have been found to produce neuronal cells (Egar and Singer, 1972; Nordlander and Singer, 1978; Arsanto et al., 1992; Benraiss et al., 1996; Zhang et al., 2000). Ependymal cells line the central canal of the spinal cord in all vertebrates, but in regenerating spinal cord they either retain some of the developmental potential of the embryonic neuroepithelium, or they can be stimulated by injury to proliferate and remodel their tissue organization (Chernoff, 1996). We show in the present study, a strong expression of nestin in the cells lining the central canal throughout the regeneration process (Figure 3G), confirming that this neuroepithelium helped reorganize the spinal cord tissue after lesion.

Although proliferation and migration of neural stem cells in the central canal of spinal cord during injuries are well studied in mammals (Horner et al., 2000; Mothe and Tator, 2005), new-born neurons from neural stem cells post SCI are very limited and vulnerable (Dobkin and Havton, 2004; Ohori et al., 2006). Moreover, recent data reveal that the ependymal stem cells eventually lose their proliferative capacity during their migration to the lesion site (Chi et al., 2006; Ke et al., 2006). In the present study, we showed that new-born neurons from both stumps of the injured spinal cord in urodele amphibians are oriented towards the lesion site giving the impression that they are migrating in order to fill the gap and form a new regenerated portion of the cord (Figure 1). We also illustrated that ependymal cells are most likely the major source of proliferative cells. Moreover, recent studies in zebra fish showed that new-born neurons were seen to be regenerated from proliferating ventricular radial glia precursors (Reimer et al., 2008, 2009; Kuscha et al., 2012a,b) and that they form synapses indicating functional integration of these cells into the nervous tissue (Adolf et al., 2006; Grandel et al., 2006; Kroehne et al., 2011; Rothenaigner et al., 2011).

In fact, an ependymal response occurs during spinal cord regeneration in all vertebrates that can regenerate injured spinal cord as adults: teleost fish, urodele amphibians and lizards in their tails (Simpson, 1968; Egar et al., 1970; Egar and Singer, 1972; Anderson et al., 1986, 1994; Alibardi and Meyer-Rochow, 1988; Duffy et al., 1992). It has been recently shown that quiescent ependymoglial cells can be activated to proliferate and regenerate the lost cells after a CNS lesion (Berg et al., 2010). As ependymal remodeling occurs, there are interactions between ependymal cells and neurons and, possibly, between ependymal cells and fibrous astrocytes and oligodendrocytes (Chernoff, 1996; Moftah et al., 2008, 2012; Fahmy and Moftah, 2010).

Glial cells have been recently demonstrated to be able to differentiate into neuronal lineages (Morrens et al., 2012), and participate in the neuronal replacement in CNS injuries (Buffo et al., 2005). During the regeneration process in urodele spinal cord, there are GFAP-positive cells (Zamora and Mutin, 1988; Fahmy and Moftah, 2010). Bodega et al. (1994) surveyed 11 vertebrate species from fish to mammals examining GFAP expression in ependymal cells. They found that lower vertebrates had more GFAP in ependymal cells than higher vertebrates. Previous studies argued that GFAP+ cells could differentiate into neurons (Garcia et al., 2004; Berninger et al., 2007; Namba et al., 2011), activate astrocytes (Fahmy and Moftah, 2010) and that GFAP+ astroglia can resume proliferation after SCI.

**Figure 7:**
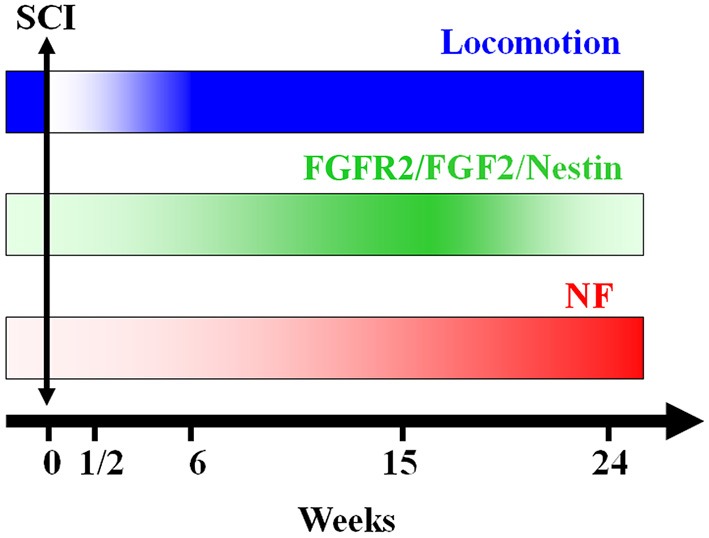
**A schematic representation of the conclusion of our findings: axonal re-growth follows a rostrocaudal direction and is related to FGF-2 and FGFR2 mRNA levels, neuronal differentiation and locomotor recovery**. FGF-2 acting through its receptor FGFR2 could be implicated in ependymal cells differentiation into **neuronal progenitors** expressing **NF** after becoming adult neurons.

The modulation of urodele ependymal cell proliferation and differentiation by bFGF demonstrates properties reminiscent of mammalian neural stem cells (O’Hara and Chernoff, 1994; Zhang et al., 2000; Temple, 2001). It is possible that, instead of retention of embryonic properties *per se*, regeneration capacity reflects expression of neural stem cell properties in specialized cell populations. As they respond to the early events following injury, ependymal cells may become a buffer between the neurons and the processes that trigger secondary cell death and axonal degeneration (Chernoff, 1996). In the present study we show that the up regulation of FGF-2 and its receptor FGFR2 is time-dependent. They increase right after lesion reaching a peak at 15 weeks post-transection, after which they decrease during locomotion recovery (Figure 7). It has been previously suggested that there is an autoregulatory mechanism by FGF-2 for producing appropriate numbers of neurons. As neurogenesis proceeds, the levels of FGF-2 increase to prevent further neuronal production from progenitors. This may be through promotion of a specific glial progenitor at the expense of the neuronal progenitor (Nelson and Svendsen, 2006).

In conclusion, the present study shows that, after complete transection of the mid-trunk spinal cord, when the animal’s hind limbs are entirely paralyzed, Nestin, FGF-2 and FGFR2 are gradually expressed throughout the regeneration process reaching a peak at 15 weeks after lesion concomitant with recovery of the locomotor activity. Following this peak, the levels of these factors diminish dramatically while the neurofilament marker NF starts to increase showing the transformation of neural progenitors into adult neurons (Figure 7). This suggests that neurogenesis is a main player in the spontaneous regeneration observed in this species.

Finally, urodele spinal cord regeneration can make an important contribution by defining the requirements for successful CNS regeneration through experimental manipulation of a regenerating adult system that allows examination of the cell interactions that elicit regeneration. Nevertheless, it is clear that we are just starting to understand the integration of various molecular regulators of neurogenesis.

## Conflict of interest statement

The authors declare that the research was conducted in the absence of any commercial or financial relationships that could be construed as a potential conflict of interest.
